# Getting insights into chemical composition and antiherpetic capability of jujube (*Ziziphus jujuba* mill.) drupes

**DOI:** 10.1016/j.heliyon.2024.e37037

**Published:** 2024-08-28

**Authors:** Annalisa Chianese, Hamid Mushtaq, Bianca Maria Nastri, Maria Vittoria Morone, Rosa Giugliano, Humaira Khan, Simona Piccolella, Carla Zannella, Severina Pacifico, Massimiliano Galdiero, Anna De Filippis

**Affiliations:** a Department of Experimental Medicine, University of Campania "Luigi Vanvitelli", 80138, Naples, Italy; b Department of Environmental, Biological and Pharmaceutical Sciences and Technologies, University of Campania "Luigi Vanvitelli", 81100, Caserta, Italy

**Keywords:** Herpesvirus, *Zizyphus jujuba* Mill., UHPLC-HR MS/MS, Polyphenols, Ursane-type triterpenes

## Abstract

Food plant diversity in bioactive compounds makes them an exploitable resource in the search for effective natural products to prevent or treat viral infections. Therefore, in the framework aimed at studying the antiviral properties of extractive mixtures from fruits (and their waste) grown in the Campania Region (Italy), jujube drupes (*Zizyphus jujuba* Mill.) were our focus. The drupes were dissected into their peel, pulp and seed parts, each of which was extracted by ultrasound-assisted maceration and further fractionated, thus obtaining, beyond the sugar fraction, a polyphenolic fraction and a lipid fraction. UHPLC-HR MS/MS tools highlighted that the polyphenolic component of the seed was strongly dissimilar from that of the edible parts, being constituted by swertisin and its derivatives. Moreover, the peel mostly accounted for triglycosylated flavonols, whereas the pulp was rich in volatile aromatic glycosides. Among lipids, *p*-coumaroyl triterpenes mainly characterized the peel. All fractions were screened for their cytotoxicity, and non-toxic concentrations of each extract were tested against herpes simplex virus type 1 (HSV-1) by plaque assays. Molecular tests and Western blot analyses were also carried out. The jujube mixtures, in detail the peel and pulp polyphenolic fractions, and peel lipophilic fraction (the latter enriched mainly in ursane-type triterpenes), showed a marked inhibitory activity against HSV-1 acting in the early stages of viral infection and preventing attachment of the virus to the host cell. The acquired data suggest jujube active mixtures as promising candidates for the prevention and treatment of herpetic lesions.

## 1 Introduction

The incidence of viral infections rose significantly worldwide, as well as the number of viral strains resistant to routinely used drugs. Most herpetic infections pathogenic to humans are due to herpes simplex viruses (HSVs), which consist of two types, i.e., HSV-1 and HSV-2, capable of inducing infections mainly (but not exclusively) in the orofacial and genital areas [1,2]. Rarely, HSV-1 induces severe diseases reaching the brain, provoking a diffuse infection resulting in herpes simplex encephalitis (HSE) [3]. Another serious disease caused by HSV-1 is neonatal herpes, associated with permanent neurological disabilities in the newborn [[4], [5], [6]]. Currently, the antiviral arsenal for treating herpetic infections is limited and includes nucleoside analogs, such as acyclovir, interfering with DNA polymerase/thymidine kinase and helicase–primase inhibitors such as amenamevir [7,8]. However, especially for severe herpetic infections, early diagnosis and therapy are imperative, and the research and identification of new compounds with different mechanisms of action and able to target a broad spectrum of viruses, should be intensified. Indeed, plant specialized metabolites are of increasing interest due to their broadly reported bioactive properties, including antimicrobial, antioxidant, anti-tumoral and anti-inflammatory [[9], [10], [11], [12], [13], [14]]. Among these compounds, polyphenols gained a lot of attention, as these compounds, which are commonly involved in defense against pathogens by destroying lipid membranes and thus altering the integrity of cellular components [15], are able also to inhibit or inactivate enzymes, leading to the microbial death. Certainly, the great structural variability of polyphenols strongly affects their bioactivity, and getting insights into the polyphenol identity appears to be mandatory for better targeting biomedical applications. In this context, three *Vitis vinifera* cvs., namely Fiano, Aglianico and Greco, were diversely extracted to achieve procyanidin or stilbenoid extracts, which showed to be active against Gram-positive bacteria and viruses [[12], [13], [14]]. Greco cv. extracts excelled for their ability against herpesviruses by targeting the early steps of infection [12]. Similarly, a *Vitis vinifera* leaf extract, mainly accounting for flavonols and flavones, was observed to exert antiviral potential against HSV-1 and the severe acute respiratory syndrome coronavirus type 2 (SARS-CoV-2) [14], while a novel polyphenol-based nutraceutical, Taurisolo [13], showed an anti-herpetic effect, directed on the viral surface. This recent and promising evidence suggests that plant mixtures with high phenolic content need to be investigated and explored as new anti-viral agents. Thus, continuing within this framework, jujube drupes (*Ziziphus jujuba* Mill.) turn into the focus.

Jujube, also called Chinese date or Chinese jujube, is one of the oldest cultivated fruit plant in the world and is the most important species of the large cosmopolitan Rhamnaceae family, given its economic, ecological, and social importance. This super-fruit [16], which is ethno-pharmacologically used for its diuretic, emollient, and laxative properties, is a rich source, beyond macro and micronutrients, of valuable non-nutritive bioactive compounds, like triterpenic acids, alkaloids, poly-phenols, and other pigments. The search on the Pubmed database showed that 1357 papers concerning plants of the genus *Zizyphus*, mainly *Z. jujuba* Mill., were published in 2003–2023. In particular, in the last five years, new research papers were aimed at highlighting the role of the fruit phytochemicals in preventing brain damage [17], counteracting anti-inflammatory diseases [18] or helping as a supplementary strategy against Covid-19 [19,20]. However, all these reports did not distinguish among the different parts of the fruits. Although an attempt was made by [21], their attention was mainly devoted to the evaluation of the antioxidant capacity of extracts from seeds, pulp, and peel, without linking the results to the identification of the bioactive chemical constituents. Instead, it is well-known that the phytochemical composition of plant organs or tissues is strongly influenced by biotic and abiotic factors, where the extraction procedure (e.g. technique, solvent polarity, experimental parameters) has a pivotal role in the enrichment of compounds of interest [22], promoting diffusive and/or osmosis processes that otherwise can deplete the plant matrix. In this context, taking into account the high saccharide content of jujube fruit, ultrasound-assisted maceration (UAM) in pure ethanol and further fractionation were performed to minimize saccharide extraction, while enhancing the recovery of specialized metabolites, mainly polyphenols and lipid components, such as triterpenes and fatty acids. Thus, deepening the chemical aspects of jujube produced in the Campania Region (Italy) paved the way to define the profile in specialized metabolites of the three parts of the fruit (peel, pulp, and seed) by an untargeted approach in high-resolution mass spectrometry while originally exploring the antiviral capability of the prepared jujube mixtures.

## 2 Materials and methods

### 2.1 Fruit collection, extraction, and fractionation

**Fig. 1:**
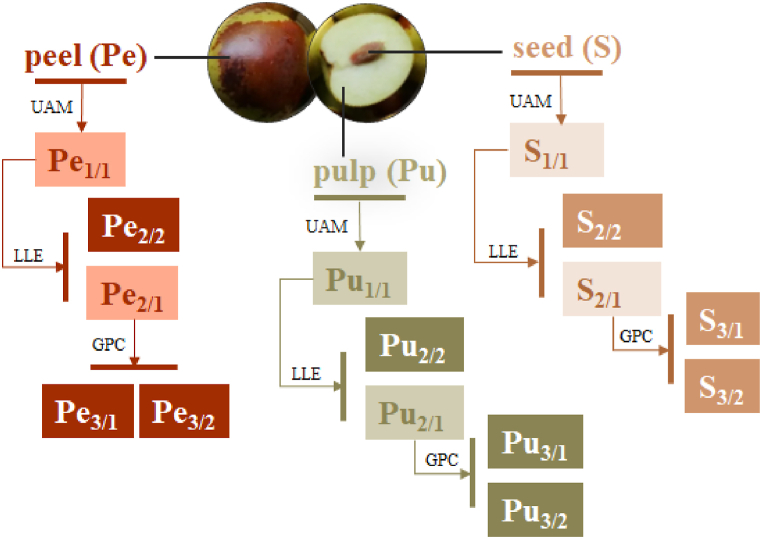
Fractionation scheme of *Zizyphus jujuba* Mill. fruit parts; Pe = peel; Pu = pulp; S = seed; UAM = Ultrasound Assisted Maceration, LLE = Liquid Liquid Extraction; GPC = Amberlite XAD-4 Gel Permeation Chromatography.

Jujube (*Zizyphus jujuba* Mill.) was collected at fruit maturity (by evaluating fruit characteristics such as size, skin, flesh color and firmness) in October 2022 in Naples (Italy) and immediately frozen at −80 °C after dissection into peel (Pe; 107.9 g), pulp (Pu; 177.9 g) and seed (S; 41.6 g). Lyophilization was carried out using the Biocool FD-1A-50 (Biocool. Beijing, China) instrument. Then, the dried matrices were powdered and extracted by UAM (Branson Ultrasonics™ Bransonic™ M3800-E, Danbury, CT, USA) using ethanol as maceration solvent in the ratio 1:4 (g of matrix:mL of solvent). Two sonication cycles were performed at 40 kHz, 30 min each. Then, the samples were filtered and dried under vacuum (Heidolph Hei-VAP Advantage, Germany), obtaining Pe1/1 (12.2 g; 11.3 % yield), Pu1/1 (11.1 g; 6.2 % yield), and S1/1 (1.48 g; 3.5 % yield) extracts, from peel, pulp, and seed, respectively. The extracts were further fractionated involving liquid-liquid extraction (LLE) using the biphasic solutions 13:7:5 (CHCl_3_:MeOH:H_2_O, *v:v:v*) for peel and seed crude extracts, and 13:7:3 (CHCl_3_:MeOH:H_2_O, *v:v:v*) for the pulp sample. Thus, the hydroalcoholic fractions Pe2/1 (8.0 g), Pu2/1 (7.8 g), and S2/1 (700 mg), and an organic fraction Pe2/2 (460 mg), Pu2/2 (137 mg), and S2/2 (385 mg), were obtained for each fruit part. The hydroalcoholic ones underwent gel permeation chromatography (GPC) on Amberlite XAD-4 polystyrene-divinylbenzene resin (h 70 cm, Ø 4.0 cm), eluting with water and MeOH. Thus, aqueous fractions Pe3/1 (7.3 g), Pu3/1 (6.9 g), and S3/1 (640 mg), and alcoholic ones Pe3/2 (57 mg), Pu3/2 (163 mg), and S3/2 (20 mg) were obtained. The fractionation steps are summarized in Fig. 1. All the fractions labeled with the subscripts 2/2, 3/1, and 3/2 were chemically profiled by UHPLC-ESI-QqTOF-MS/MS and evaluated for their bioactivity.

### 2.2 UHPLC-ESI-QqTOF-MS/MS analyses

The UHPLC method was carried out on a NEXERA UHPLC system (Shimadzu, Tokyo, Japan), using a Luna® Omega C18 column (50 × 2.1 mm i.d., 1.6 μm particle size; Phenomenex, Torrance, CA, USA) for lipophilic (2/2) and polyphenolic fractions (3/2), whereas a Luna® Omega SUGAR 100 Å (100 × 2.1 mm, 3 μm; Phenomenex, Torrance, CA, USA) was employed for aqueous fractions (3/1). In all the experiments, the mobile phase consisted of water and acetonitrile, both acidified with 0.1 % formic acid, with a 0.5 mL/min flow rate and a sample injection volume of 2 μL. The detailed UHPLC elution gradient is reported in supplementary materials (Table S1).

HRMS analyses were performed by using the AB SCIEX TripleTOF® 4600 spectrometer (AB Sciex, Concord, ON, Canada), equipped with a DuoSpray™ ion source operating in negative electrospray (ESI) ion mode. The APCI probe was used for automated mass calibration in all scan functions using the Calibrant Delivery System (CDS). An untargeted approach was developed, consisting of a full scan TOF survey, and eight information-dependent acquisition (IDA) MS/MS scans. Detailed HR mass spectrometry parameters are reported in supplementary materials (Table S2). The instrument was controlled by Analyst® TF 1.7 software, while data processing was carried out using PeakView® software version 2.2.

### 2.3 Cell line and virus

The kidney epithelial cells of the African green monkey, named Vero 76 (ATCC CRL-1587, CVCL-0603), were grown in Dulbecco's Modified Eagle Medium (DMEM; Gibco; Thermo Fisher Scientific, Waltham, MA, USA) with 4.5 g/L glucose, 2 mm L-glutamine, 100 IU/mL penicillin/streptomycin solution and 10 % fetal bovine serum (FBS; Gibco; Thermo Fisher Scientific, Waltham, MA, USA) in a humidified atmosphere with 5 % CO_2_ at 37 °C. We confirmed that Vero 76 cells used in this work have been authenticated within the last three years; additionally, we also tested them using a mycoplasma detection kit (Mycoplasma PCR Detection Kit, Cat. No. G238, abm, New York, United States) and confirmed they were mycoplasma free. HSV-1 (strain SC16) was propagated on Vero 76 cells as previously reported [23].

### 2.4 Cytotoxicity assay

Cytotoxicity of jujube extracts was evaluated by 3-(4,5-Dimethylthiazol-2-yl)-2,5-Diphenyltetrazolium Bromide (MTT) assay (Sigma-Aldrich, St. Louis, MO, USA) as reported elsewhere [24]. Vero 76 (2 × 10^4^ cells/well) were seeded in a 96-well plates and incubated at 37 °C and 5 % CO_2_. The day after, cell monolayer was exposed to different concentrations of jujube extracts ranging from 200 to 25 μg/mL for 24 h (h). After incubation, the medium was removed and 0.5 mg/mL of MTT solution was added to each well for 3 h. Formazan crystals were then solubilized by DMSO for 10 min (min) and cell viability was evaluated by measuring absorbance at 570 nm by TEKAN M − 200 reader (Tecan, Männedorf, Switzerland). Cytotoxicity of jujube extracts was expressed as a percentage (%) of cell viability according to the following formula:

% cell viability = (Absorbance of treated sample)/(Absorbance of untreated cell (CTRL+)) × 100

Untreated cells were used as positive control (CTRL+), while DMSO 100 % as negative control (CTRL−).

### 2.5 Antiviral assays

The antiviral activity was investigated by plaque reduction assays as described elsewhere [25,26]. Vero 76 (1.4 × 10^5^ cells/well) were plated in 24-well plates and incubated at 37 °C and 5 % CO_2_ for 24 h. To better understand in which stage of viral infection the jujube extracts were active, four different treatments were conducted. a) Co-treatment: HSV-1 at a multiplicity of infection (MOI) of 0.01 and non-cytotoxic concentrations of jujube extracts were inoculated simultaneously on cell monolayer for 1 h at 37 °C; b) Virus pre-treatment: virus at MOI of 0.1 and jujube extracts were incubated together for 1 h at 37 °C. After that, the mixture (virus/extract) was inoculated on cell monolayer for 1 h; c) Cell pre-treatment: jujube extracts were first placed on cell monolayer for 1 h, and then cells were infected with HSV-1 at MOI of 0.01 for 1 h; d) Post-treatment: cell monolayer was first infected with the virus at MOI of 0.01 for 1 h, and after exposed to jujube extracts for another hour at 37 °C. At the end of each treatment, Vero 76 cells were washed with citrate buffer and incubated with DMEM supplemented with 10 % FBS and 3 % carboxymethylcellulose (CMC), (Sigma-Aldrich, St. Louis, MO, USA) for 48 h. Cells were fixed with 4 % formaldehyde (Sigma-Aldrich) and stained with 0.5 % crystal violet (Sigma-Aldrich). Viral plaques were counted, and the % of viral inhibition was calculated according to the following formula:

% of viral inhibition = (1- (plaques counted in treated cells)/(plaques counted in infected cells (CTRL -))) × 100

### 2.6 Real-Time PCR and Western-blot analyses

The antiviral activity of jujube extracts was also investigated by Real-Time PCR and Western-blot [23]. Virus pre-treatment was performed as described above. Thirty hours after infection, RNA and protein extract were collected using TRIzol reagent (Thermo Fisher, Waltham, MA, USA) and lysis buffer, respectively. After that, RNA was retro-transcribed in cDNA by BlasTaq TM 2X qPCR MasterMix (Applied Biological Materials, Richmond, Canada) and Real-time PCR was performed to analyze expression levels of the gene UL27. The relative target threshold cycle (Ct) was normalized to glyceraldehyde 3-phosphate dehydrogenase (GAPDH) used as a housekeeping gene. In Table 1, the primer sequences are reported.

**Table 1:** Forward and reverse sequences of primers used in Real-time PCR.

Gene	Forward sequence	Reverse sequence
UL-27	GCCTTCTTCGCCTTTCGC	CGCTCGTGCCCTTCTTCTT
GAPDH	CCTTTCATTGAGCTCCAT	CGTACATGGGAGCGTC

Protein lysate was quantified by spectrophotometer and, after denaturation, 40 μg of proteins were loaded. After transfer to nitrocellulose membrane and blocking in 3 % Bovine Serum Albumin (BSA, Bio-Rad) for 1 h, membrane was incubated with anti-tubulin (1:1000; Elabscience) and anti-gB (1:5000; Elabscience), followed by secondary incubation with anti-mouse (1:10,000; Elabscience) and anti-rabbit (1:5000; Elabscience), respectively. Proteins were detected with ECL Western blotting detection system (Bio-Rad) and quantified via ImageLab 6.1 software. Bands were normalized to tubulin.

### 2.7 Statistical analysis

All experiments were carried out in triplicate and expressed as mean ± Standard Deviation (SD) calculated by GraphPad Prism (version 8.0.1; Software for 2D graphing and statistics; GraphPad Software Inc.: San Diego, CA, US, 2018; www.graphpad.com [27]). One-way ANOVA followed Dunnett's multiple comparisons test was performed; a value of *p* ≤ 0.0001 was considered significant.

## 3 Results and discussion

### 3.1 HRMS-based characterization of polyphenolic fractions (Pe_3/2_, Pu_3/2_ and S_3/2_)

Flavonoid glycosides are the representative compounds in all parts of the fruit investigated, although their relative content in the peel fraction (Pe_3/2_) appeared 3.74-fold higher than in the pulp sample (Pu_3/2_). The extract from the seeds (S_3/2_) showed a total amount relatively comparable to the peel extract, although the identified metabolites differed. The TOF-MS data of the identified compounds in these fractions are listed in Table 2.

**Table 2:** TOF-MS data of compounds in polyphenolic fractions (Pe_3/2_, Pu_3/2_ and S_3/2_). RDB = Ring Double Bonds.

A	RT	[M − H]^-^*m/z* found	Formula	RDB	Error (ppm)	Tentative assignment
1	3.047	431.1565	C_19_H_28_O_11_	6	1.4	zizybeoside I
2	3.619	577.1360	C_30_H_26_O_12_	18	1.5	procyanidin (isomer 1)
3	4.203	431.1565	C_19_H_28_O_11_	6	1.4	zizybeoside I (isomer)
4	4.555	577.1366	C_30_H_26_O_12_	13	2.5	procyanidin (isomer 2)
5	5.036	401.1440	C_18_H_26_O_10_	6	2.7	benzylprimeveroside
6	5.113	449.1095	C_21_H_22_O_11_	11	1.3	dihydrokaempferol 7-*O*-hexoside
7	5.114	595.1688	C_27_H_32_O_15_	12	2.1	naringenin 6,8-di-*C*-hexoside
8	5.309	771.1993	C_33_H_40_O_21_	14	0.5	quercetin dihexose deoxyhexose (isomer 1)
9	5.455	771.1995	C_33_H_40_O_21_	14	0.7	quercetin dihexose deoxyhexose (isomer 2)
10	5.720	755.2017	C_33_H_40_O_20_	14	−1.6	kaempferol 3-*O*-hexoside-7-*O*-deoxyhexosylhexoside
11	5.963	593.1519	C_27_H_30_O_15_	13	1.2	apigenin 6,8-di-*C*-hexoside
12	6.036	755.2027	C_33_H_40_O_20_	14	−3.1	kaempferol 3-*O*-deoxyhexosylhexoside-7-*O*-hexoside
13	6.329	741.1897	C_32_H_38_O_20_	14	1.8	quercetin 3-*O*-deoxyhexosylhexoside-7-*O*-pentoside
14	6.503	593.1525	C_27_H_30_O_15_	13	2.2	saponarin
15	6.546	771.2000	C_33_H_40_O_21_	14	1.4	quercetin dihexose deoxyhexose (isomer 3)
16	6.725	771.2006	C_33_H_40_O_21_	14	2.2	quercetin dihexose deoxyhexose (isomer 4)
17	6.739	563. 1410	C_26_H_28_O_14_	13	0.7	apigenin 6-*C*-pentoside-8-*C*-hexoside
18	6.853	623.1616	C_28_H_32_O_16_	13	−0.1	chrysoeriol 6,8-di-*C*-glucoside
19	6.952	741.1900	C_32_H_38_O_20_	14	2.2	quercetin 3-*O*-pentosylrutinoside
20	7.207	593.1518	C_27_H_30_O_15_	13	1.0	apigenin *C*-(*O*-hexosyl)hexoside
21	7.337	607.1680	C_28_H_32_O_15_	13	1.9	isospinosin
22	7.375	919.2470	C_42_H_48_O_23_	19	−4.7	(hexosyl)vanilloylspinosin
23	7.431	609.1486	C_27_H_30_O_16_	13	4.1	quercetin 3-*O*-deoxyhexosylhexoside (I)
24	7.503	889.2379	C_41_H_46_O_22_	19	−3.3	(hexosyl)benzoylspinosin
25	7.581	609.1480	C_27_H_30_O_16_	13	3.1	quercetin 3-*O*-deoxyhexosylhexoside (II)
26	7.584	607.1677	C_28_H_32_O_15_	13	1.4	spinosin
27	7.639	477.0686	C_21_H_18_O_13_	13	2.4	quercetin 3-*O*-hexuronide
28	7.672	725.1953	C_32_H_38_O_19_	13	3.1	kaempferol 3-*O*-pentosyl deoxyhexosylhexoside
29	7.773	597.1825	C_27_H_34_O_15_	11	3.5	phloretin 3′,5′-di-*C*-hexoside
30	7.784	945.2654	C_44_H_50_O_23_	20	−1.7	(hexosyl)feruloylspinosin
31	7.846	445.1160	C_22_H_22_O_10_	12	4.4	swertisin
32	7.999	593.1521	C_27_H_30_O_15_	13	1.5	kaempferol 3-*O*-deoxyhexosylhexoside (I)
33	8.054	755.2057	C_33_H_40_O_20_	14	2.2	kaempferol 7-*O*-(hexosyl)deoxyhexosylhexoside
34	8.228	649.1769	C_30_H_34_O_16_	14	−0.8	acetylspinosin
35	8.409	593.1526	C_27_H_30_O_15_	13	2.5	kaempferol 3-*O*-deoxyhexosylhexoside (II)
36	8.648	579.1374	C_26_H_28_O_15_	13	3.2	quercetin 3-*O*-pentosyldeoxyhexoside (I)
37	8.819	579.1371	C_26_H_28_O_15_	13	2.7	quercetin 3-*O*-pentosyldeoxyhexoside (II)
38	9.225	813.2232	C_39_H_42_O_19_	19	−1.9	sinapoylspinosin
39	9.382	783.2138	C_38_H_40_O_18_	19	−1.0	feruloylspinosin
40	10.700	869.2858	C_43_H_50_O_19_	19	−1.8	phaseoylspinosin

Aromatic glycosides are also part of the three fractions, representing 12.7 %, 29.5 %, and 10.1 % of Pe_3/2_, Pu_3/2_, and S_3/2_, respectively. Benzyl glycosides, differing in the saccharide moiety, were tentatively identified. Compound **5**, with the deprotonated molecular ion at *m/z* 401.1440 was likely benzyl primeveroside. Supporting this hypothesis, following the loss of a pentosyl unit, the ion at *m/z* 269.1036 was formed [28], while the ion at *m/z* 161.0379 consisted in the dehydrated hexose (Fig. S1). The two isomers **1** and **3**, with deprotonated molecular ions at *m/z* 431.1565, are benzyldisiloxane derivatives. In particular, the benzyl sophoroside (**1**), namely zizybeoside I, was previously reported as jujube constituent [29]. Compound **3** was likely a gentiobiosyl derivative of benzyl alcohol. The TOF MS/MS spectra of both compounds yielded fragment ions [M-H-162]^-^ at *m/z* 269.09 and [M-H-162-108]^-^ at *m/z* 161.04 (Fig. S1). The other identified compounds, as described above, are flavonoid *O*- and C-glycosides. Compound **6**, which is most abundant in S_3/2_ and Pu_3/2_ fractions, was putatively identified as the dihydrokaempferol 7-*O*-glucoside. In fact, in the TOF-MS/MS spectrum the aglycone ion at *m/z* 287.06 was detected, together with the ions at *m/z* 259.06 and *m/z* 269.05, generated from the latter after the loss of CO and water, respectively (Fig. S2). Dihydrokaempferol was previously isolated from the root of *Zizyphus jujuba* Mill. var. *spinosa* [30].

**Fig. 2:**
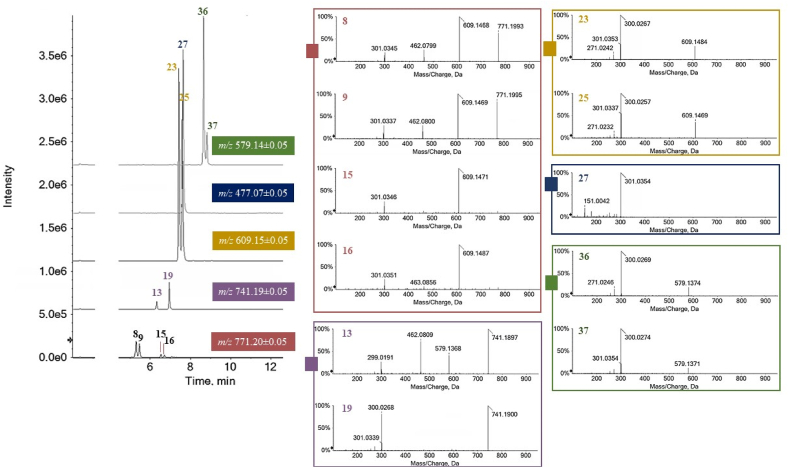
XICs (eXtracted Ion Chromatograms) of the ions at *m/z* 771.20, 741.19, 609.15, 477.07 and 579.14 due to jujube quercetin glycosides. The TOF-MS/MS spectra are also reported.

Quercetin *O*-glycosides were among the main compounds (Fig. 2), and were identifiable based on their typical neutral losses.

Quercetin glucuronide (**27**) with [M − H]^-^ ion at *m/z* 477.0686 was the mono-glycoside, whereas glycone moieties consisting in two residues were in compounds **23**, **25**, **36** and **37**, and a trisaccharide portion was in compounds **8**, **9**, **13**, **15**, **16**, **19**. In particular, the disaccharide derivatives **23** and **25**, sharing the neutral loss of 308 Da to achieve the aglycone ion, were in accordance with rutin and its isomer, likely quercetin 3-*O*-robinobioside (quercetin 3-rhamnosyl-(1 → 6)-galactoside) [31]. Rutin was the first flavonol found in jujube leaves [32] and is considered a bitterness indicator in jujube fruit [33]. Based on literature data, compounds **36** and **37**, whose deprotonated molecular ions lost 278 Da according to a dehydrated pentosyl-deoxyhexose, were tentatively identified as quercetin 3-*O*-arabinorhamnoside and quercetin 3-*O*-xylorhamnoside, respectively [34]; Fig. 2. Compounds **8**, **9**, **15**, **16** were isomers, whose saccharidic part accounted for two hexose residues and one deoxyhexose. The first two, eluting at shorter retention times, were differently abundant in the three fruit parts investigated, while the last two were present only in the peel. Finally, the saccharidic moiety of compounds **13** and **19** consisted in a deoxyhexose, a hexose and a pentose, so much so that in the TOF-MS/MS spectrum of compound **19** a loss of 440.16 Da was observed. This latter neutral loss was also observed for compound **28**, which was one of the four kaempferol trisaccharides identified in jujube extracts, together with compounds **10**, **12** and **33**, which differed from the previous one as their sugar moiety was due two dehydrated hexoses and one deoxyhexose (Fig. S3). The kaempferol disaccharides **32** and **35** were distinguishable based on the relative abundance of the aglycone ion, suggesting two isomers in which the glycosylation site is at C-3 aglycone carbon (**32**) or at C-7 (**35**).

Several C-glycosides were detected, and identified thanks to characteristic neutral losses due to internal cleavages within the sugar rings (Fig. S4). Among these, compound **7**, which constitutes 17 % and 16 % of the Pe_3/2_ and Pu_3/2_ fractions, respectively, was likely naringenin 6,8-di-*C*-glucoside. Compounds **11**, **17**, and **18** were other di-*C*-glycosyl compounds, while compound **14** has been tentatively identified as saponarin, an apigenin *O*,*C*-diglucoside [21]. Compound **20** was a *C*-(*O*-glucosyl) glucoside of apigenin, which also was the aglycone of compounds **11** and **17**. These three compounds shared a common deprotonated molecular ion, but differed significantly in their TOF-MS/MS spectra. In particular, the presence of the ion at *m/z* 431.0996 in the spectrum of compound **14**, also known as 7-*O*-(β-d-glucosyl)isovitexin, highlighted the loss of a dehydrated hexose as *per* an O-glycosidic bond. For compound **20**, the loss of 180 Da suggested the loss of the sugar residue from the hydroxymethyl function of the C-linked glucose. Compound **17** has been tentatively identified as a *C*-pentoside-*C*-hexoside derivative of apigenin. Confirming this hypothesis, literature data highlight the isolation of apigenin 6-*C*-α-l-arabinopyranoside-8-*C*-β-d-glucopyranoside [35]. Compound **18** was the di-*C*-glucosyl derivative of chrysoeriol (*i.e.* 3′-methoxy luteolin). The dihydrochalcone phloretin 3,5-di-*C*-glucoside (**29**) has been identified in all parts of the fruit, although it appears to be more abundant in the peel.

**Fig. 3:**
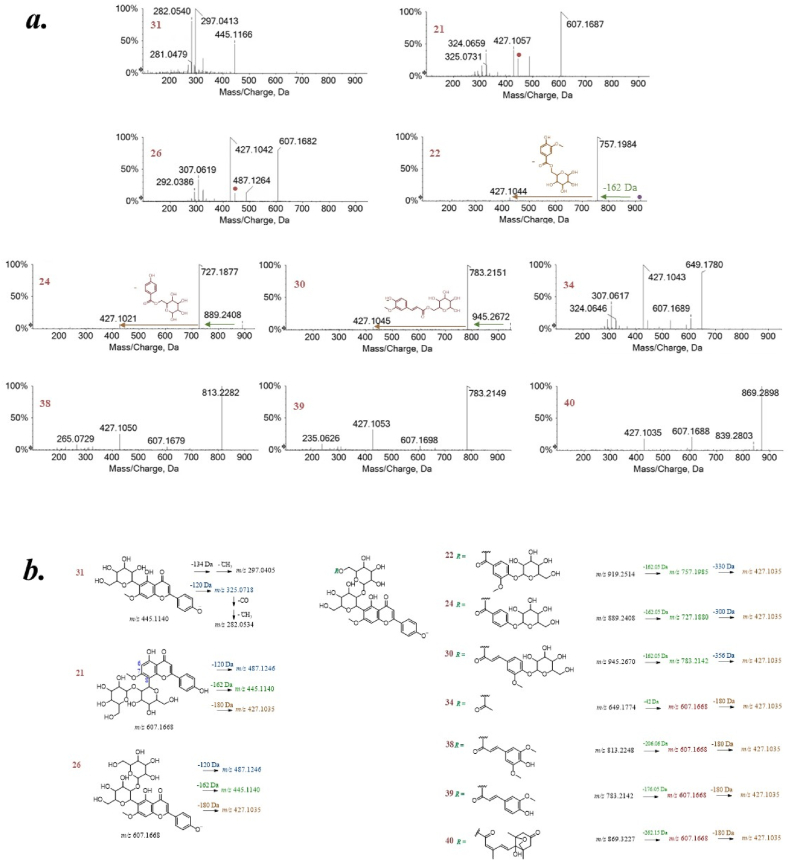
TOF-MS/MS spectra (a) and hypothesized fragmentation patterns (b) of swertisin (**31**) and its derivatives from jujube seeds. The theoretical *m/z* values are reported below each structure.

All the other identified compounds were detected only in the S_3/2_ fraction, and are derivatives of swertisin (Fig. 3). This latter was also identified, hereinafter labeled as compound **31**, based on its deprotonated molecular ion at *m/z* 445.1160. Swertisin, a *C*-glucosylflavone, is known for its antidiabetic, anti-inflammatory, and antioxidant effects, and also for its being an antagonist of the adenosine A1 receptor [36]. Among its derivatives, the isomers isospinosin (**21**) and spinosin (**26**) were identified. Spinosin, previously isolated from the seeds of *Ziziphus jujuba* var. *spinosa*, has been characterized as 2″-β-*O*-glucopyranosyl swertisin. Spinosin may aid the sleep mechanism and interact with 5-hydroxytryptophan and 5-hydroxytryptamine 1A receptors [37]. It has also been reported that subchronic low dose spinosin application for 2 weeks significantly increases latency time in the passive avoidance task in healthy adult rodents. Spinosin reverses cognitive impairment, improves memory and learning, and reduces Aβ1-42 oligomers in the brain of AD-induced mice. Compounds **22**, **24**, **30**, **34**, **38**–**40** are acylated derivatives of spinosin. Compound **22**, with a deprotonated molecular ion at *m/z* 919.2470, has been tentatively identified as (hexosyl)vanilloylspinosin (Fig. 3) [38].

Compound **24**, with a deprotonated molecular ion at *m/z* 889.2379, corresponded to 6‴-(4‴’-O-β-D-glucopyranosyl)-benzoyl-spinosin, previously reported from the seeds of *Ziziphus mauritiana* (Rhamnaceae) [39]. Other acylated *C*-glycosyl flavonoids, 6‴-(−)-phaseoylspinosin (**40**), 6‴-sinapoylspinosin (**38**), and 6‴-feruloylspinosin (**39**), were also previously isolated from the seeds of *Z. mauritiana* [40]. Furthermore, the glucosylated derivative of feruloylspinosin, never reported before in the literature, was also putatively identified. Finally, based on the TOF-MS/MS spectrum, compound **34** was putatively acetylspinosin. In fact, the deprotonated molecular ion lost 42 Da (acetyl moiety) to provide the ion at *m/z* 607.17, attributable to spinosin. Lastly, in S_3/2_ fraction two deprotonated molecular ions at *m/z* 577.14 (**2** and **4**) were detected, whose TOF MS/MS spectra were consistent with two procyanidins B-type (Fig. S5). Both fragmented giving rise to characteristic product ions through the quinone methide (QM) cleavage pathway of the interflavanoid bond, as well as by heterocyclic ring fission (HRF) and retro-Diels-Alder (RDA) cleavage within the heterocyclic ring of one of the two subunits [41]. The relative quantification, obtained by considering the areas under each peak in their respective XICs (Extracted Ion Chromatograms), evidenced that jujube polyphenols were differently distributed within the three jujube drupe parts (Fig. S6). When the data were plotted considering the percentage of each compound in its relative fraction, it was highlighted that quercetin triglycosides were mainly abundant in the peel fraction, volatile glycosylated compounds were more representative of the pulp component, while swertisin-derivated compounds made the seed fraction unique.

### 3.2 HRMS-based characterization of lipid fractions (Pe_2/2_, Pu_2/2_ and S_2/2_)

The organic fractions (Pe_2/2_, Pu_2/2_ and S_2/2_) resulting from liquid-liquid extraction were characterized by the presence of lipidic components with a wide structural variety, and differentially abundant in the three parts of the jujube fruit.

Triterpenes represent one of the most representative classes in the *Ziziphus* genus, attracting significant attention in recent scientific literature [[42], [43], [44]]. Specifically, for *Z. jujuba*, more than 120 triterpenoids have been identified to date, in the form of aglycones, esters, ethers, and glycosides. The aglycone triterpenes, despite having a pentacyclic skeleton composed of 30 carbon atoms, can be distinguished into different subclasses related to lupane, oleanane, and ursane [45]. Table 3A summarizes the UHPLC-TOF/MS data related to the tentatively identified triterpenes. The limited fragmentation in tandem mass spectrometry experiments and the unavailability of commercial standards for individual metabolites belonging to this class for comparing retention times in UHPLC have made it challenging to confirm the identity. Therefore, identification is based almost exclusively on the molecular formula and the elution order in reverse-phase chromatography of isomeric compounds. The compound with the formula C_30_H_48_O_3_ (*m/z* 455.3552; **T14**) can be attributed to ursolic acid. Compounds **T15** and **T16** likely correspond to oleanonic and ursonic acids, in which the OH substituent at C-3 is replaced by a ketonic group [39]. The presence of the ketonic function in ursonic acid, identified as one of the main compounds in ripe fruits, seems responsible for various enhanced biological properties compared to those exhibited by ursolic acid (e.g. antihyperglycemic, anti-inflammatory, antiviral, antitumor) [20].

**Table 3:** TOF-MS data of A) triterpenes, and B) fatty acids and their derivatives tentatively identified in lipid fractions (Pe_2/2_, Pu_2/2_ and S_2/2_). RDB = Ring Double Bonds.

A	RT	[M − H]^-^*m/z* found	Formula	RDB	Error (ppm)	Tentative assignment
T1	5.018	485.3274	C_30_H_46_O_5_	8	0.3	ceanothic acid
T2	5.622	471.3478	C_30_H_48_O_4_	7	−0.4	alphitolic acid
T3	5.713	471.3495	C_30_H_48_O_4_	7	3.2	maslinic acid
T4	5.841	631.3660	C_39_H_52_O_7_	14	3.1	*p*-coumaroyl (epi)ceanothic acid 1
T5	5.976	633.3816	C_39_H_54_O_7_	13	3.0	*p*-coumaroyl tormentic acid
T6	5.979	469.3340	C_30_H_46_O_4_	8	3.5	zizyberanalic acid
T7	6.045	631.3651	C_39_H_52_O_7_	14	1.7	*p*-coumaroyl (epi)ceanothic acid 2
T8	6.061	485.3274	C_30_H_45_O_5_	8	0.3	epiceanothic acid
T9	6.301	631.3664	C_39_H_52_O_7_	14	3.8	*p*-coumaroyl (epi)ceanothic acid 3
T10	6.310	469.3337	C_30_H_46_O_4_	8	2.9	zizyberanalic acid isomer
T11	6.848	617.3877	C_39_H_54_O_6_	13	4.8	*p*-coumaroyl alphitolic acid
T12	6.934	617.3876	C_39_H_54_O_6_	13	4.6	*p*-coumaroyl maslinic acid
T13	6.925	471.3493	C_30_H_48_O_4_	7	2.8	pomolic acid
T14	7.238	455.3552	C_30_H_48_O_3_	7	4.7	ursolic acid
T15	7.829	453.3392	C_30_H_46_O_3_	8	3.9	oleanonic acid
T16	7.918	453.3372	C_30_H_46_O_3_	8	−0.5	ursonic acid
B	**RT**	**[M-H]**^**-**^***m/z* found**	**Formula**	**RDB**	**Error (ppm)**	**Tentative assignment**
F1	0.941	201.1137	C_10_H_18_O_4_	2	2.3	decanedioic acid
F2	1.540	215.1291	C_11_H_20_O_4_	2	1.0	undecanedioic acid
F3	2.132	227.1291	C_12_H_20_O_4_	3	1.0	dodecenedioic acid
F4	2.402	229.1444	C_12_H_22_O_4_	2	−0.6	dodecanedioc acid
F5	3.097	243.1602	C_13_H_24_O_4_	2	0.1	tridecadienoic
F6	3.887	257.1759	C_14_H_26_O_4_	2	0.3	Tetradecanedioic
F7	0.953	301.2017	C_16_H_30_O_5_	2	−1.2	hydroxy-hexadecanedioic acid 1
F8	1.419	301.2018	C_16_H_30_O_5_	2	−0.8	hydroxy-hexadecanedioic acid 2
F9	1.543	303.2174	C_16_H_32_O_5_	1	−1.0	trihydroxy-hexadecanoic acid 1
F10	2.011	303.2176	C_16_H_32_O_5_	1	−0.3	trihydroxy-hexadecanoic acid 2
F11	2.230	329.2332	C_18_H_34_O_5_	2	−0.4	trihydroxy-octadecenoic acid 1
F12	2.323	303.2172	C_16_H_32_O_5_	1	−2.0	trihydroxy-hexadecanoic acid 3
F13	2.832	329.2334	C_18_H_34_O_5_	2	0.2	trihydroxy-octadecenoic acid 2
F14	3.522	241.1812	C_14_H_26_O_3_	2	1.2	oxo-tetradecanoic acid
F15	4.651	269.2126	C_16_H_30_O_3_	2	1.4	oxo-hexadecanoic acid 1
F16	4.786	269.2127	C_16_H_30_O_3_	2	1.8	oxo-hexadecanoic acid 2
F17	4.959	285.2074	C_16_H_30_O_4_	2	0.9	oxo-hydroxy-hexadecanoic acid 1
F18	5.129	285.2071	C_16_H_30_O_4_	2	−0.1	oxo-hydroxy-hexadecanoic acid 2
F19	5.386	295.2281	C_18_H_32_O_3_	3	0.8	hydroxyoctadeca-dienoic acid
F20	5.714	297.2435	C_18_H_34_O_3_	2	−0.1	oxo-octadecanoic acid 1
F21	5.885	297.2435	C_18_H_34_O_3_	2	−0.1	oxo-octadecanoic acid 2
F22	5.961	313.2393	C_18_H_34_O_4_	2	2.8	dihydroxy-octadecanoic acid
F23	7.775	279.2336	C_18_H_32_O_2_	3	2.3	Linoleic acid
F24	8.217	255.2334	C_16_H_32_O_2_	1	1.7	Palmitic acid
F25	8.433	281.2488	C_18_H_34_O_2_	2	0.7	Oleic acid
F26	5.151	571.2899	C_25_H_49_O_12_P	2	1.8	LPI (16:0)
F27	5.408	452.2776	C_21_H_44_NO_7_P	1	−1.5	LPE (16:0)
F28	5.623	483.2719	C_22_H_45_O_9_P	1	−2.0	PG (16:0)
F29	6.215	409.2356	C_19_H_39_O_7_P	1	−0.9	LPA (16:0)
F30	6.259	599.3197	C_27_H_53_O_12_P	2	−0.8	LPI (18:0)

The deprotonated molecular ion at *m/z* 471.349(8) was in accordance with the presence of alphitolic acid (**T2**), maslinic acid (**T3**), and pomolic acid (**T13**), previously described as components of the leaves and fruits of *Z. jujuba* [46,47]. The esterification at C-3 of these metabolites with a *p*-coumaric acid residue led to the formation of compounds **T11** and **T12** with a deprotonated molecular ion at *m/z* 617.3877(6). Although the fragmentation was limited also in this case, the TOF-MS/MS spectrum showed a fragment ion at *m/z* 145.03, corresponding to the dehydrated hydroxycinnamic acid portion, confirming its presence (Fig. S7(A)). Similarly, esterification can occur with ceanothic or epiceanothic acid (**T1** and **T8**; *m/z* 485.3274), leading to the formation of metabolites **T4** and **T9** at *m/z* 631.366(5) (C_39_H_52_O_7_, RDB = 14) (Fig. S7(B)). These ones, along with the hypothetical hydroxycinnamoyl derivative of tormentic acid (**T5**; *m/z* 633.3816) (Fig. S7(C)), were detected only in the epicarp of the fruit. Finally, the deprotonated molecular ion at *m/z* 469.334 for triterpenes with Rt = 5.979 and 6.310 min, consistent with the molecular formula C_30_H_46_O_4_, were attributed to two isomers of ziziberanalic acid (**T6** and **T10**). Ziziberanalic acid differs from (epi)ceanothic acid for a formyl group instead of a carboxyl group. This hypothesis is supported by the absence of decarboxylation, characteristic of the TOF-MS/MS spectrum of ceanothic acid (Fig. S7). The composition of free fatty acids in the investigated organic fractions includes molecules with a carbon skeleton of 16 or 18 carbon atoms, exhibiting different unsaturation and hydroxylation degrees. In Table 3B UHPLC-TOF/MS data related to the tentatively identified fatty acids and derivatives are reported. The composition and content of fatty acids in jujube fruits can be influenced by both their cultivars and the production areas due to differences in climate, soil, water, and sunlight, as well as the fruit's maturation state and harvesting time [32,48]. Beyond palmitic acid (**F24**; *m/z* 255.2334), the presence of the polyunsaturated fatty acids linoleic (**F23**; *m/z* 279.2336) and oleic (**F25**; *m/z* 281.2488) was detected, along with their oxygenated derivatives, concentrated mainly in the fruit mesocarp.

In the case of the compound **F26** with the empirical formula C_25_H_49_O_12_P, palmitic acid was esterified to glycerol, which in turn was linked to a phosphate group and an inositol residue. A similar structure, with the only difference being the presence of stearic acid (18:0) instead of palmitic acid, was hypothesized for the compound **F30**. In both cases, a neutral loss of inositol (180 Da) from the deprotonated molecular ion was observed, along with two diagnostic fragments at *m/z* 152.9962(1) and 315.049, whose structures are depicted in Fig. S8. The fragment ion at *m/z* 152.99 was also present in the TOF-MS/MS spectra of metabolites **F28** (C_22_H_45_O_9_P; *m/z* 483.2719) and **F29** (C_19_H_39_O_7_P; *m/z* 409.2356). The rationalization of the fragmentation patterns led to the putative identification of these molecules as PG (16:0) and LPA (16:0), respectively [49]. TOF-MS/MS spectra and proposed structures are reported in Fig. S8.

Lastly, palmitic acid is also part of the compound **F27** with a deprotonated molecular ion at *m/z* 452.2776, tentatively identified as LPE (16:0). In this case, the glycerophosphate moiety binds an ethanolamine residue, highlighted by the diagnostic fragment at *m/z* 196.0385 (C_5_H_11_NO_5_P^−^, exact mass *m/z* 196.0380) [50], and its structure is depicted in Fig. S8.

The tissue-specific accumulation was highlighted also for lipid constituents (Figura S8). In particular, triterpenes were mainly found in Pe_2/2_ fraction, whose content in keto derivatives of oleanolic and ursolic acid (**T15** and **T16**) was estimated to be equal to 39.5 %. Ursolic acid (**T14**) appeared to be relatively more abundant in seed fraction than in edible components, together with oleic and linoleic acids. All other lipid representatives were primarily concentrated in Pu_2/2_. Cluster analysis highlighted the diversity in triterpenes of peel fraction, which segregated from the other two components, while highlighting that seed extract was in a separate group when fatty acids and their derivatives were considered (Fig. S9).

### 3.3 HRMS-based characterization of sugar fractions (Pe_3/1_, Pu_3/1_ and S_3/1_)

The aqueous fractions resulting from GPC on Amberlite XAD-4 consisted of two hexose monosaccharides (fructose and glucose) and the disaccharide sucrose [51]. The identification was carried out by comparison with commercial standards analyzed under the same experimental conditions as the samples under study (Fig. S10a). The abundance of drupe saccharides required fractionation procedures that better unmasked the bioactive components, mainly flavonoid glycosides, triterpenes and fatty acid derivatives. Although particularly abundant in the pulp, the relative abundance of sucrose in the sugar fraction of the three parts highlights its equal distribution, where fructose and glucose decrease moving from the peel to the seed (Fig. S10b).

### 3.4 Cytotoxicity of jujube fraction towards Vero 76 cells

**Fig. 4:**
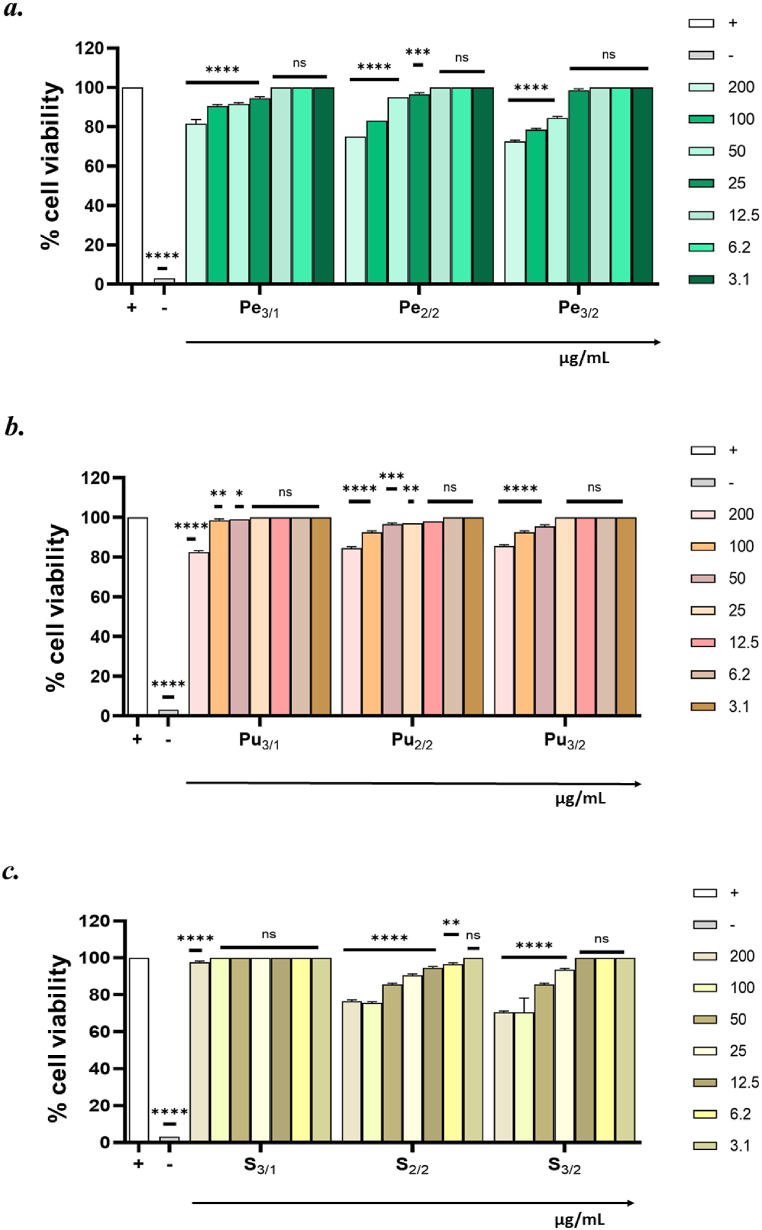
Cell viability evaluation. The viability of Vero 76 cells was analyzed 24 h after exposure to different concentrations of ***a.*** peel (Pe, green-colored), ***b.*** pulp (Pu, orange-colored), and ***c.*** seed (S, yellow-colored) fractions. Dimethyl sulfoxide (DMSO) was used as negative control (−), while non-treated cells were used as positive control (+). ****p < 0,0004; ***p < 0,0010; **p < 0,0073; *p < 0,0421; ns: non-significant. (For interpretation of the references to color in this figure legend, the reader is referred to the Web version of this article.)

Before exploring the antiviral potential of jujube extracts, their cytotoxicity was analyzed via MTT assay. This test was employed to measure cell viability as it assesses the reduction of the tetrazolium salt MTT to formazan by the mitochondrial dehydrogenases occurring only in viable cells. Different concentrations of jujube fractions were tested within the range from 200 to 3.1 μg/mL. Cell viability was quantified in respect to untreated cells (CTRL+). No toxicity was observed at all the tested concentrations for all the fractions of peel, seed and pulp (Fig. 4). Our results are in agreement with the data reported by Rajaei et al. They evaluated the cytotoxic effect of *Z. jujuba* on several cellular systems confirming no toxicity also at the highest concentrations tested [52].

### 3.5 Antiviral activity of the extracts against HSV-1

To explore the antiviral activity of the constituted jujube fractions and their potential mechanism of action, plaque reduction assays against HSV-1 was carried out, using four distinct treatment schemes: co-treatment, virus pre-treatment, cell pre-treatment and post-treatment. All fractions were tested at not cytotoxic concentrations, i.e., in the range 200–3.1 μg/mL for all of them.

**Fig. 5:**
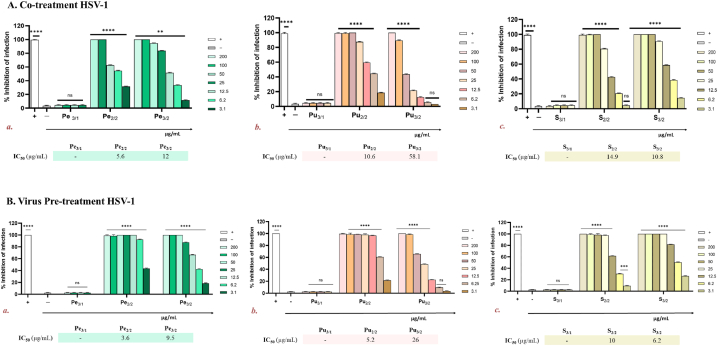
Antiviral activity of jujube extracts against HSV-1 infection in **A.** co-treatment assay: ***a.*** peel fraction (Pe); ***b.*** pulp fraction (Pu), and ***c.*** seed extracts (S). Infected cells were used as negative control (−), while cells infected and treated with an extract with noted anti-HSV-1 activity [14] represented the positive control (+). Dunnet's multiple comparison test: ****p < 0.0001; **p < 0.0073; *p < 0.0336; ns: non-significant. **B.** Virus pre-treatment assay was performed: ***a.*** peel fraction (Pe); ***b.*** pulp fraction (Pu), and ***c.*** seed extracts (S). Infected cells were the negative control (−), while virus treated with an extract with noted anti-HSV-1 activity (PMID: 34209556) represented the positive control (+). Dunnet's multiple comparison test: ****p < 0,0001; ***p = 0,0002; ns: non-significant.

As shown in Fig. 5A, when the extracts and virus were inoculated on cell monolayer simultaneously (co-treatment), the sugar fraction, mainly accounting of sucrose, fructose and glucose, was the only one not active in inhibiting HSV-1 infection regardless of the nature of extracts. A very strong inhibitory potential was detected for both lipid and polyphenolic fractions, as also evidenced by the half-maximal inhibitory concentration (IC_50_) (Fig. 5A). In particular, it was observed that lipid fraction was the most active compared to the polyphenolic one for both peel and pulp, meanwhile S_3/2_, which is the only one, among the three polyphenolic fractions, constituted by swertisin and its derivatives, exhibited a higher antiviral activity respect to S_2/2_.

When virus was pre-treated with extracts (virus pre-treatment), an improvement of the antiviral effect was present (Fig. 5B). The trend was very similar to that observed in co-treatment assay, therefore lipid and polyphenolic fractions were the most active. Among all and similarly to co-treatment results, Pe_2/2_ fraction showed the best activity with an IC_50_ at 3.6 μg/mL, followed by Pu_2/2_ (IC_50_ = 5.2 μg/mL) and S_3/2_ (IC_50_ = 6.2 μg/mL). These data suggested that the active fractions interact directly with the viral particles blocking the early stages of viral infection.

Similar results have also been observed with several extracts, such as *Vitis vinifera* leaf extract, elderberry extract, green tea extract, and others, confirming that extracts rich in polyphenols reduce the viral infection targeting the viral surface [14,53,54]. On the other side, no reduction in infection was observed in cell pre-treatment and post-treatment assays, indicating that the extracts could neither interact with the cell surface nor interfere with intracellular targets or infection stages (Fig. S11). This indicates that the mechanism of action of these compounds may be specifically due to interactions that occur during the initial stages of viral infection or when the virus is directly exposed to jujube fractions. Altogether our findings demonstrate that *Z. jujuba* extract is endowed of an antiviral potential acting in the extracellular phase of infection, at the moment in which the virus attaches to the cellular membrane. No precise mechanism has been elucidated, but, probably, the extract may damage the viral particle interfering with the subsequent step of penetration inside the host cell.

### 3.6 Evaluation of viral gene expression

Considering the higher activity of extract Pe_2/2_, Pu_2/2_ and S_3/2_ (in descending order), further investigations were carried out to confirm their antiviral effect. Real-Time PCR analysis was performed to assess the extracts impact on the expression of a critical gene associated with HSV-1 viral entry, namely UL27. This gene is a late gene playing a crucial role during the first stages of the viral life cycle: UL27 gene encodes the envelope glycoprotein B (gB), a class III fusion protein crucial for viral membrane and host cell membrane fusion [55].

**Fig. 6:**
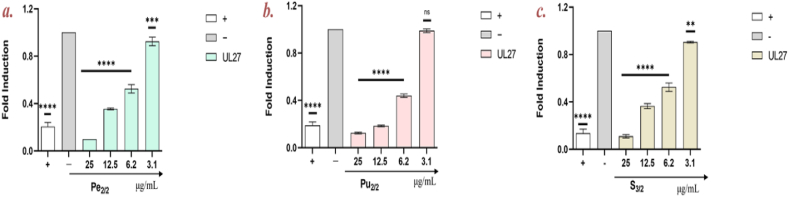
Molecular assay. Real-time PCR was performed to evaluate the effect of ***a.*** peel fraction (Pe); ***b.*** pulp fraction (Pu), and ***c.*** seed extracts (S) on viral gene expression. The expression of HSV-1 UL27 was analyzed. Infected cells were the negative control (−), while virus treated with an extract with noted anti-HSV-1 activity [14] represented the positive control (+). Dunnet's multiple comparison test: ****p < 0.0001; ***p = 0.0008; **p = 0.0010; ns: non-significant.

As previously reported, the extract Pe_2/2_ was the most active: as indicated in Fig. 6, the expression level of UL27 was very low up to 6.2 μg/mL. Very similarly, Pu_2/2_ significantly reduced the gene expression in the range of concentrations 25–6.2 μg/mL. On the contrary, S_3/2_ showed a lower ability in inhibiting the infection: UL27 was detectable already at 12.5 μg/mL, according to data obtained by plaque assays (Fig. 5, panels A and B).

### 3.7 Western blot analyses

**Fig. 7:**
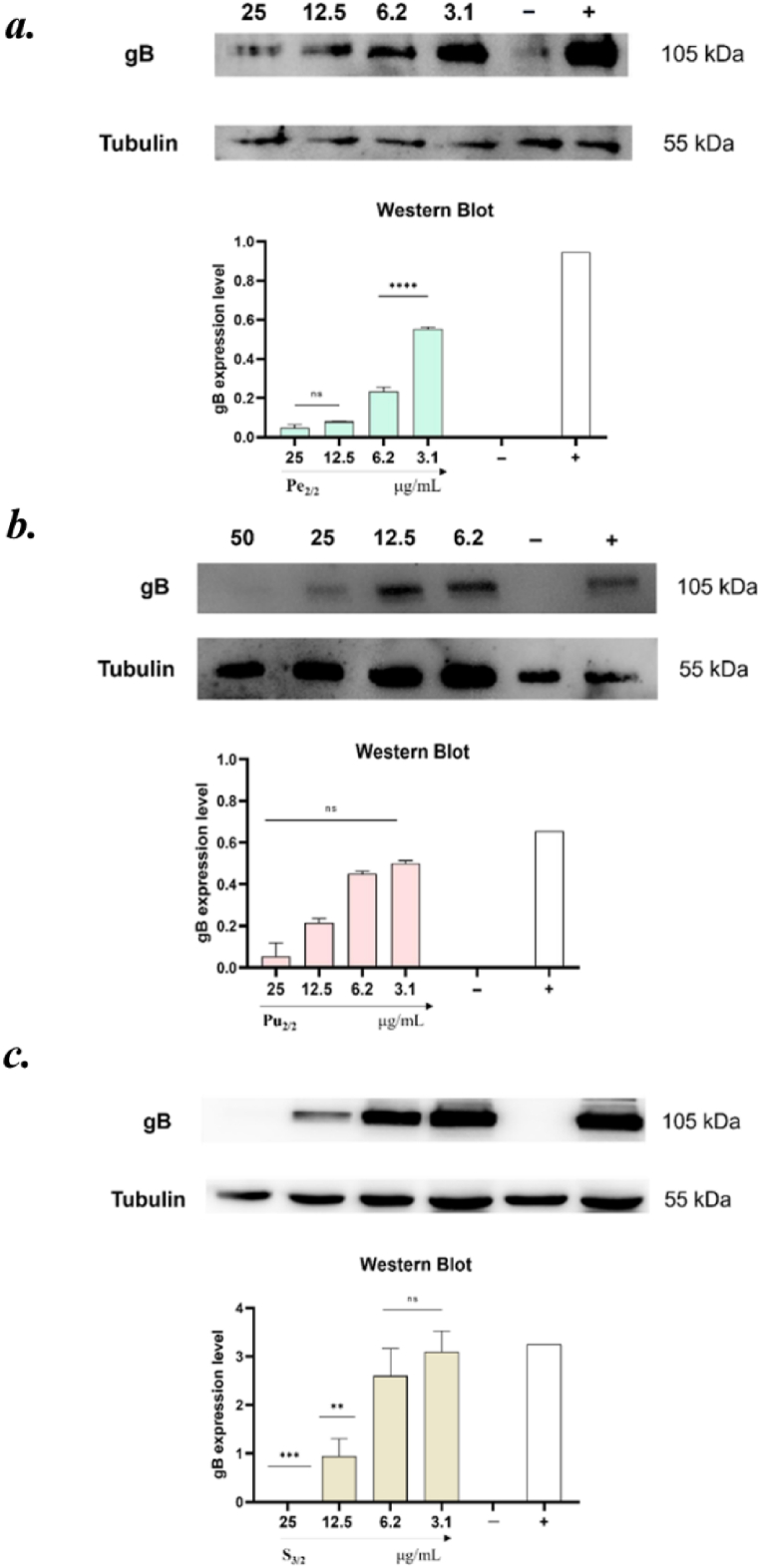
Western blot analyses. The assay was performed to evaluate the effect of ***a.*** peel fraction (Pe); ***b.*** pulp fraction (Pu), and ***c.*** seed extracts (S) on gB protein expression. Non-treated cells were used as negative control (−), while infected cells were used as positive control (+). The amount of gB was compared to that of tubulin. Dunnet's multiple comparison test: ****p < 0.0001; ***p = 0.0003; **p = 0.0017; ns: non-significant.

The antiviral activity of Pe_2/2_, S_3/2_ and Pu_2/2_ was further investigated via another molecular analysis. Western blot was employed to assess the extracts ability to reduce the expression of gB at translation level (Fig. 7). A strong reduction of gB protein level was observed, confirming both data obtained through plaque assays and Real-time analysis. We demonstrated that Pe2/2 was the extract with the best antiviral activity and was able to reduce by double gB protein production at 6.2 μg/mL; on the other side, Pu2/2 and S3/2 resulted active until 25 μg/mL in decreasing protein level.

Thus, lipid fractions from the different parts of *Ziziphus jujuba* Mill., i.e., peel, seed and pulp were demonstrated for the first time as antiherpetic promising agents. To date, several studies have been conducted and only report the antiviral effect of compounds isolated from jujube fruit. Hong et al. [56] described that betulinic acid blocked influenza A/PR/8 virus infection at 50 μM, while in another study, authors showed that three compounds derived from jujube roots were endowed with antiviral potential against the porcine epidemic diarrhea virus (PEDV) [57]. Indeed, among the triterpenes identified in peel lipid fraction, ursolic acid (**T14**), which accounted for 13 % of the prepared fraction, when isolated from *Mallotus peltatus* and tested at 9.0 μg/mL exhibited nearly 100 % inhibition against HSV-1, also inhibiting plaque formation at 80 % level [58]. The antiviral activity of ursolic acid was broadly tested towards different viruses: it was observed to counteract human immunodeficiency virus (HIV-1) protease, to be efficacious against chronic hepatitis C (HCV), to be able to interfere with rotavirus replication cycle, and to acts as selective inhibitor of papilloma virus [59]. Analogously, although it was less investigated than ursolic acid, its keto derivative, ursonic acid (**T16**), was found to suppress HSV-1 and HSV-2 cytopathic effects, and its inhibitory effect was ten times higher than that exhibited by ursolic acid. This finding is in line with our data on peel lipid fraction, in which ursonic acid is the most abundant compound followed by oleanonic acid (**T15**). While searching on jujube antiviral polyphenols, interesting data were found in relation to two purified methoxylated flavones were active against tobacco mosaic virus replication [60]. Quercetin and some different its derivatives were also widely screened [61]. In particular, quercetin showed to inhibit the expressions of HSV proteins such as glycoprotein D (gD) and Infected Cell Protein 0 (ICP0), and also to suppress TLR-3 dependent inflammatory responses in infected cells [62].

This evidence suggests that constituted jujube fractions could be favorably employed, also considering the established traditional prescription, named Yakammaoto, which consists of 9 ingredients, including jujube fruit, widely used in China for the management of airway symptoms due to its high effect in inhibiting picornavirus infection [63,64].

## 4 Conclusions

Recently, a great deal of interest in the medical field has been aroused by natural extracts [65]. As reported in several studies, polyphenols particularly flavonoids, are known for their anti-inflammatory, antimicrobial, anticancer, antioxidant and antiviral properties [66]. In this study, we investigated the inhibitory potential of jujube drupe extracts against HSV-1, identifying the jujube fruit as a promising candidate for further pharmaceutical applications.

Considering the high sugar content of jujube fruit, with the aim to deplete sugar constituents in favor of the specialized metabolites, ultrasound-accelerated maceration in pure ethanol was carried out [67]. This led to the acquisition of alcoholic extracts from each part of the fruit (seed, pulp and peel), further fractionated to create a polyphenolic extract and a lipid extract. The extracts thus obtained were chemically profiled through ultra-high performance liquid chromatography coupled with high resolution mass spectrometry. Finally, fractions were tested for their antiviral effect. Lipid and polyphenol fractions showed a strong antiherpetic activity, which could be due to their ability to target the viral membrane damaging it and hindering the adsorption of virus on the host cell. We found out that the peel lipid fraction, whose triterpene component accounted for 13 % in oleanonic acid and 26.4 % of ursonic acid, was the most active. On the contrary, as expected, the aqueous fractions, containing only sucrose, glucose and fructose, exhibited no antiviral effect.

## Funding

Funding for this research was provided by Enforcing the THERapeutic Arsenal againSt Emerging and Reemerging RNA Viruses Prin 2022_Fondi Prin_2022W97H54, CUP: B53D23003630006.

## Data availability

Data will be made available on request.

## CRediT authorship contribution statement

**Annalisa Chianese:** Writing – original draft, Methodology, Investigation. **Hamid Mushtaq:** Methodology, Investigation. **Bianca Maria Nastri:** Methodology, Investigation, Data curation. **Maria Vittoria Morone:** Methodology, Investigation, Data curation. **Rosa Giugliano:** Visualization. **Humaira Khan:** Methodology, Investigation. **Simona Piccolella:** Writing – review & editing. **Carla Zannella:** Writing – original draft, Validation, Supervision. **Severina Pacifico:** Writing – review & editing, Supervision, Project administration. **Massimiliano Galdiero:** Writing – review & editing, Supervision, Project administration, Conceptualization. **Anna De Filippis:** Writing – review & editing, Supervision, Project administration, Conceptualization.

## Declaration of competing interest

The authors declare that they have no known competing financial interests or personal relationships that could have appeared to influence the work reported in this paper.
