# Supplementary material for: Getting insights into chemical composition and antiherpetic capability of jujube (Ziziphus jujuba mill.) drupes
Source: Heliyon. 2024 Aug 28;10(17):e37037. doi: 10.1016/j.heliyon.2024.e37037 (PMC11402244; doi:10.1016/j.heliyon.2024.e37037)
Supplement: Multimedia component 1 [file mmc1.docx]

**Supplementary materials**

**Table 1S.** UHPLC elution gradient set up for lipid (2/2), polyphenolic (3/2), and sugar (3/1) fractions

| **Lipid fraction (2/2)** | |  | **Polyphenolic fraction (3/2)** | |  | **Sugar fraction (3/1)** | |
| --- | --- | --- | --- | --- | --- | --- | --- |
| t (min) | % CH_3_CN |  | t (min) | % CH_3_CN |  | t (min) | % CH_3_CN |
| 0 | 30 |  | 0 | 5 |  | 0 | 75 |
| 1 | 30 |  | 1 | 5 |  | 12 | 75 |
| 4 | 50 |  | 7.5 | 20 |  |  |  |
| 14 | 70 |  | 9 | 20 |  |  |  |
| 15 | 95 |  | 12 | 95 |  |  |  |

**Table 2S.** High-resolution mass spectrometry (HRMS) parameters and applied potentials

| parameter | Lipid fraction (2/2) | Polyphenolic fraction (3/2) | Sugar fraction (3/1) |
| --- | --- | --- | --- |
| TOF-MS *m/z* range  accumulation time | 100 - 1300 Da  250 ms | 100 - 1500 Da  250 ms | 150 - 1500 Da  250 ms |
| TOF-MS/MS *m/z* range  accumulation time | 80-1100 Da  100 ms | 80-1300 Da  100 ms | 100-1350 Da  100 ms |
| curtain gas (CUR) | 35 psi | 35 psi | 35 psi |
| nebulizer gas (GS1)  heated gas (GS2) | 60 psi  60 psi | 60 psi  60 psi | 60 psi  60 psi |
| Ion spray voltage (ISVF) | - 4,5 kV | - 4,5 kV | - 4,5 kV |
| interface heater temperature (TEM) | 500° C | 600° C | 600° C |
| declustering potential (DP) | - 80 V | - 80 V | - 70 V |
| collision energy (CE) | - 45 kV | - 45 kV | - 35 kV |
| CE spread | 25 | 25 | 10 |

**Figure S1.** TOF-MS/MS spectra of compounds **1**, **3** and **5**. The theoretical *m/z* value is reported below the benzylhexose ion structure.

**Figure S2.** TOF-MS/MS spectrum of compound **6** and enlarged section. The fragmentation pattern is proposed and the theoretical *m/z* values are reported below each structure.

**Figure S3.** TOF-MS/MS spectra of kaempferol glycoside in jujube extracts and observed neutral losses (n.l.)

**Figure S4.** TOF-MS/MS spectra of *C,C*-diglycosides (**7**, **11**, **17**, **18**, **29**) and *O,C*-diglycosides (**14**, **20**) in jujube extracts and observed neutral losses. The theoretical *m/z* values are reported below each structure.

**Figure S5.** TOF-MS/MS spectra and hypothesized fragmentation pattern of B-type procyanidins **2** and **4**. The theoretical *m/z* values are reported below each structure.

**Figure S6.** Heatmap of the relative percentage occurrence of polyphenols in jujube fractions.


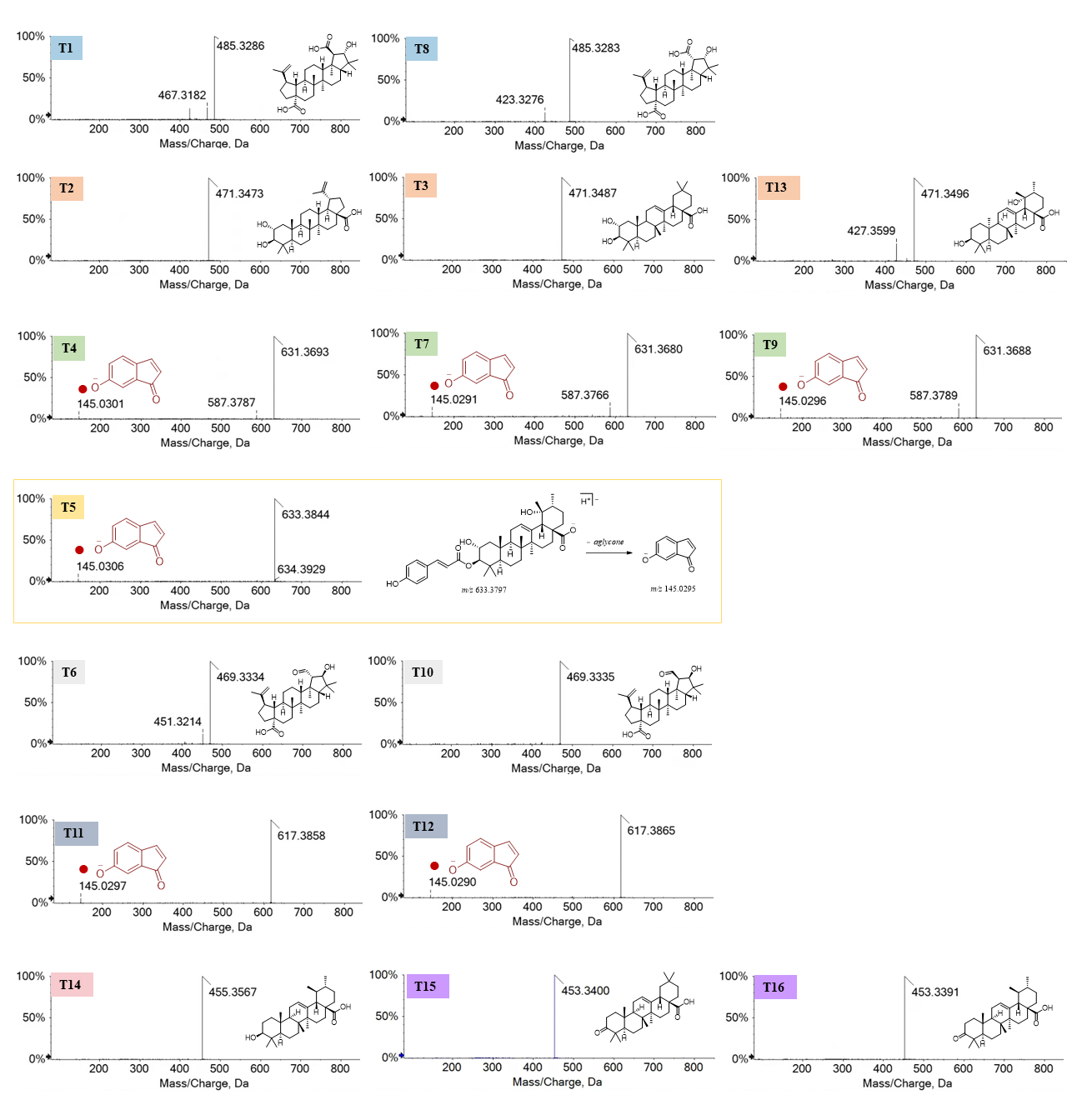


**Figure S7.** TOF-MS/MS spectra of triterpenes tentatively identified.

**Figure S8.** TOF-MS/MS spectra of **F26**-**F30**.

**Figure S9**. Heatmap of the relative percentage occurrence of ***a.*** triterpenes, and ***b.*** fatty acids and their derivatives in jujube lipid fractions.

**Figure S10.** Representative TOF-MS spectrum of peel aqueous fraction, and relative content of fructose, glucose and sucrose in the aqueous fractions of each jujube fruit part (Pe_3/1_, Pu_3/1_, and S_3/1_).


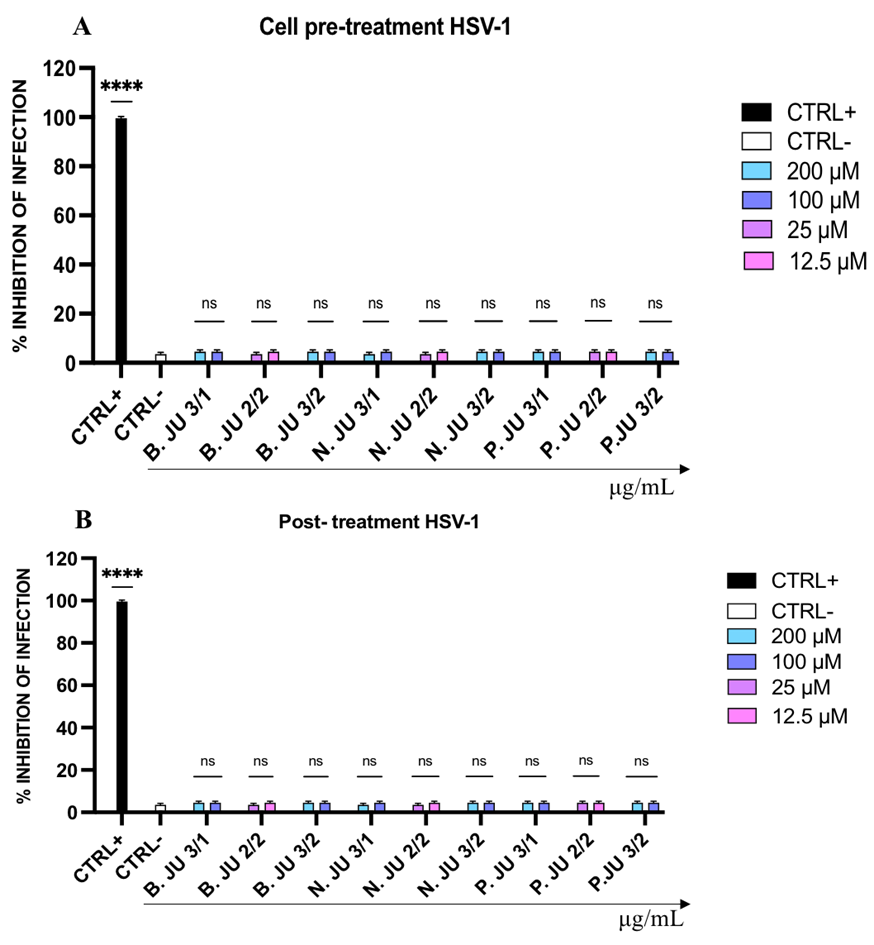


**Figure S11.** Antiviral activity levels against the HSV-1. Different assays were performed to explore the antiviral activity of the extracts against HSV-1: (A) Cell pre-treatment; (B) Post-treatment. Non-treated cells were used as positive control (CTRL +) while infected cells were used as negative control (CTRL -). Dunnet’s multiple comparison test: *ns: non-significant.*
